# SMARCA4-deficient undifferentiated uterine sarcoma: A case report and literature review

**DOI:** 10.1097/MD.0000000000043828

**Published:** 2025-08-15

**Authors:** Yu Wang, Ying Sun, Yuxin Jiang, Xing Chen, Huihua Dai

**Affiliations:** a Department of Gynecology, The First Affiliated Hospital with Nanjing Medical University, Nanjing, China.

**Keywords:** malignant tumors of the endometrium, SMARCA4, SMARCA4-deficient undifferentiated uterine sarcoma

## Abstract

**Rationale:**

SMARCA4-deficient undifferentiated uterine sarcoma (SDUS) is a rare, sporadic malignant tumor of mesenchymal origin of the uterus, which has highly aggressive and poor prognosis. In this case, we described a malignant tumor formed in the uterus occurring in a SMARCA4 deletion type and discussed its clinical characteristics, differential diagnosis, treatment and related literature analysis.

**Patient concerns:**

A 61-year-old patient underwent diagnostic scraping at a local hospital for irregular vaginal bleeding for 2 months. The postoperative pathology suggested endometrial malignancy. A subsequent pelvic ultrasound at our hospital revealed that the endometrium was not clear, and a slightly hypoechoic mass of 7 + cm was seen in the uterine cavity. This mass had an irregular shape and unclear demarcation from the myometrium. Furthermore, the presence of abundant blood flow signals was detected in and around the uterine cavity.

**Diagnoses:**

The patient was ultimately diagnosed with stage IB SDUS by postoperative routine pathology and immunohistochemistry.

**Interventions:**

The patient underwent a single-port laparoscopic total hysterectomy, bilateral adnexectomy and pelvic lymph node dissection.

**Outcomes:**

The patient has undergone 3 cycles of postoperative platinum-based chemotherapy combined with docetaxel and epirubicin, and during the follow-up period, the patient remained in good overall condition without evidence of disease progression.

**Lessons:**

SDUS is a rare uterine sarcoma which cannot be underestimated and warrants careful clinical follow-up and histological evaluation.

## 1. Introduction

SMARCA4 (SWI/SNF related BAF chromatin remodeling complex subunit ATPase 4)-deficient undifferentiated uterine sarcoma (SDUS) is a newly recognized malignant neoplasm of mesenchymal derived from the uterus. Only 30 cases have been reported worldwide, and complete clinical data are available for only 18 of them. Due to the extremely low incidence, its clinical manifestations, histomorphology and immunophenotype are challenging to distinguish from other uterine tumors, and therefore it is highly susceptible to misdiagnosis. This article describes the diagnostic process, differential diagnosis and literature analysis for a case of SDUS. Written and signed informed consent was obtained from the patient.

## 2. Case presentation

**Figure 1.**
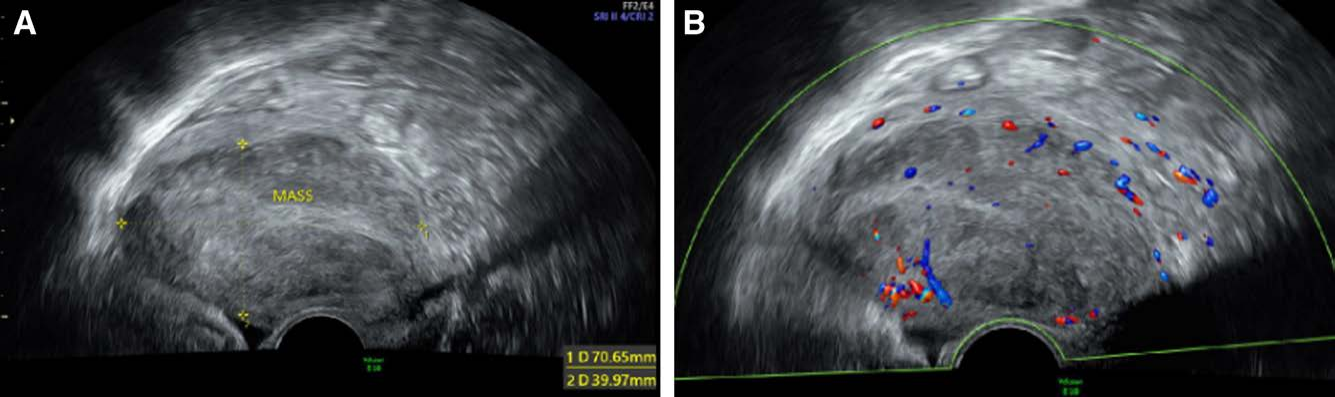
Imaging of SMARCA4-deficient undifferentiated uterine sarcoma. (A) The endometrium was indistinct, and a 71 × 40 × 54 mm slightly hypoechoic mass was seen in the uterine cavity, with an irregular shape and poorly defined borders with the myometrium. (B) Abundant blood flow signals were detected within and surrounding the uterine cavity.

The patient was 61 years old, menopausal for 10 years, with G2P2. She consulted a local hospital because of intermittent, irregular vaginal bleeding for 2 months in February 2024. And the enhanced CT of the lower abdomen showed that the uterus was enlarged, the uterus wall was unevenly thickened, the uterine cavity was filled with isodense foci, and the level of the upper edge of the cervix was involved downward. The thickened area measured approximately 24 mm in diameter, and it demonstrated noticeable heterogeneous enhancement. The thickened area was about 24 mm in diameter and showed noticeable inhomogeneous after enhancement. Gynecologic tumor markers were all normal. After hysteroscopy at an outside institution, postoperative pathology suggested endometrial malignancy, mixed epithelial-mesenchymal tumors, a tendency to high-grade adenosarcoma with overgrowth of sarcoma (undifferentiated endometrial sarcoma and undifferentiated uterine sarcoma), and small foci of carcinosarcoma, which could not be excluded entirely. Immunohistochemistry suggested ER (+), PR (+), and P53 (overexpression). She came to our hospital on March 22, 2024. Pelvic ultrasonography showed that the endometrium was unclear, and a 71 × 40 × 54 mm slightly hypoechoic mass was seen in the uterine cavity, with an irregular shape, poorly demarcated from the myometrium and with uneven internal echogenicity, and flocculent and slightly high echogenicity, with abundant blood flow signals detected in and around the mass (Fig. 1). The myometrium was poorly demarcated in the localization, with the myometrium being about 5.3 mm thicker.

After completing a preoperative evaluation to exclude surgical contraindications, the patient underwent a single-port laparoscopic total hysterectomy, bilateral adnexectomy and pelvic lymph node dissection on March 26, 2024. Intraoperatively, the uterus was grossly intact with a smooth surface, and bilateral adnexa was atrophied with no obvious abnormality in appearance, and no apparent enlarged lymph nodes were identified in the pelvis or along the abdominal aorta, and no ascites were noted, and the surface of the diaphragmatic apex, liver, stomach, spleen, intestinal tubes, and the greater omentum was smooth with no apparent tumor foci, and part of the greater omentum adhered to the abdominal wall. At the same time, the sigmoid colon had extensively adherence to the left pelvic wall and the left pelvic funnel ligament.

**Figure 2.**
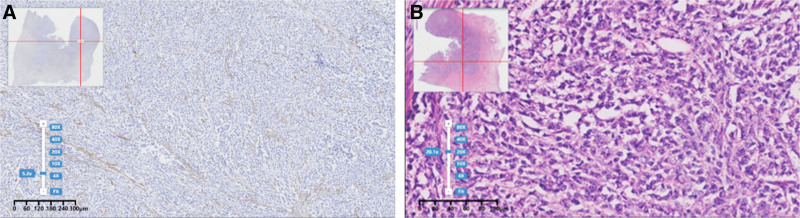
Pathological features of SMARCA4-deficient undifferentiated uterine sarcoma: (A) Tumor cells with absent expression of SMARCA4 (BRG-1) and positive expression of background uterine remnant muscle bundles (B) Diffusely uniform cells, epithelioid or spindle-shaped, without clear differentiation, with active mitotic activity.

Postoperative routine pathology suggested a malignant tumor of the uterus, and the tumor invaded the whole layer of the uterine wall, and a vascular cancer embolus was seen. Leucosomal tissue was seen in bilateral ovaries, bilateral fallopian tubes showed chronic inflammation, no tumor metastasis was seen in pelvic lymph nodes, and no malignant tumor cells were seen in ascites. Immunohistochemistry showed: tumor cells CK-pan (−), INI-1 (+), BCOR (−), BRG-1 (−), Vimentin (+), Ki67 (hot spot about 30%+),p53 (foci+), Desmin (−), H-caldesmon (−), CD34 (−), CD10 (−), Napsin A (−), MYoD1 (−), and MYoD1 (−) (Fig. 2). The patient was ultimately diagnosed with stage IB SDUS. The patient has undergone 3 cycles of postoperative platinum-based chemotherapy combined with docetaxel and epirubicin, and during the follow-up period, the patient remained in good overall condition without evidence of disease progression.

## 3. Discussion

The SMARCA4 gene encodes the BRG-1 protein, which is a subunit of the SWI/SNF chromatin remodeling complex and functions as a tumor suppressor. Deletion or inactivation of SMARCA4 is associated with the development of tumors across various organ systems, including the gastrointestinal tract, sinuses, thorax, and genitourinary organs.^[1]^ Among them, in the female reproductive system, tumors caused by SAMRCA4 deletion are more common in the ovary.^[2]^ SDUS was first reported by Kolin et al^[3]^ as an extremely rare and highly aggressive uterine sarcoma, which mainly occurs in young patients (median age 34 years, range from 23 to 62 years) with a median survival of 7 to 9 months.^[4]^ To date, fewer than 30 cases have been reported worldwide, and detailed clinical data are available for only 18 of these patients.

Based on previous literature reports, most patients with SDUS present at an advanced stage with distant metastasis disease. Lymph node metastasis was found in approximately 50% of patients at the time of diagnosis, and only about 19% of cases are diagnosed at an early stage(stage I or II).^[5]^ Clinically, most patients presenting symptoms are irregular vaginal bleeding, and masses of the uterus, cervix, and vagina may be detected on physical examination and imaging.

Like other types of endometrial malignant tumors, SDUS lacks specificity, and definitive diagnosis relies primarily on postoperative pathologic examination. Microscopically, SDUS is characterized by a prominent rhabdomyosiform pattern of epithelioid macrocells in sheets or vesicular composition, with large areas of necrosis visible. The characteristic immunohistochemical profile includes a lack of SMARCA4 (BRG-1) protein expression, along with negativity for CK-pan (−), INI-1 (+), BCOR (−), and Vimentin (−). The tumor cells also typically show negativity or only focal positivity for other epithelial markers like cytokeratin, EMA, and claudin-4, as well as mesenchymal markers such as CD10.

It has been pointed out that about 37% of undifferentiated and differentiated endometrial carcinomas (UDEC) also demonstrate biallelic inactivation of SMARCA4 and SMARCA2 and can exhibit a rhabdomyosiform morphology similar to that of SDUS.^[6]^ However, SDUS is typically negative for epithelial markers, whereas more than 80% of UDEC express keratins.^[4]^ Additionally, Next-Generation Sequencing has shown that, unlike SDUS, UDEC is usually MSI-H and mutations associated with endometrial cancer, such as PTEN and TP53. Another entity that needs to be distinguished from SDUS is small cell carcinoma of the ovary, hypercalcemia type (SCCOHT). Both SDUS and SCCOHT share similarities in their aggressive clinical behavior and lack of SMARCA4 expression. However, SCCOHT typically occurs at a younger age, presents with abdominal/pelvic masses and adnexal involvement, and less frequently manifests with vaginal bleeding or cervical masses.^[7]^ The immunophenotypic and molecular features of SDUS are almost identical to those of SCCOHT except for differentiation by the primary focus. Sixty per cent of SCCOHT patients present with comorbid hypercalcemia, whereas SDUS calcium is usually within the normal range.

SDUS is an extremely rare and rapidly disease, and there is no consensus on treatment. Among the 18 patients with available dates (see Table 1), all but 3 (83%, 15/18) had no access to surgery. The standard procedure for uterine sarcoma is total hysterectomy and bilateral adnexectomy. In general, bilateral adnexa are not preserved for highly malignant uterine sarcomas. Of the 15 operated patients, 14 (93%, 14/15) underwent a total hysterectomy and bilateral adnexectomy, of which 40% (6/15) underwent resection of the greater omentum, 40% (6/15) underwent lymph node dissection, which consisted mainly of pelvic, pelvic, and low para-aortic and retroperitoneal lymph nodes, and 13% (2/15) underwent appendiceal resection. Overall survival did not differ significantly among these patients. However, of the 2 patients who underwent appendectomy, 1 patient died 43 months after surgery, and 1 patient had no recurrence after 6 months of follow-up. Therefore, whether prophylactic appendectomy contributes to prolonged survival must be further verified. However, one of them had systemic metastases at the time of discovery, so gemcitabine and docetaxel combination chemotherapy was used to prolong survival, and her bleeding symptoms improved after 3 cycles of follow-up. Unfortunately, the ultimate outcome for this patient is unknown.

**Table 1:** Clinical data of SMARCA4-deficient undifferentiated uterine sarcoma were reported in the literature.

Reference	Age	Surgical procedure	Radiotherapy status	Follow-up time (months)	Prognosis
Kolin, 2017	25	A + B + C	N	7	I
	33	A + C	N	9	I
	34	N	N	1	I
	29	N	N	4	I
	58	A + C + D	N	43	I
Gao, 2023	27	A	Epirubicin + isocyclophosphamide	6	R
Kihara, 2021	59	A	N	12	R
Kurokawa, 2021	51	A + E	Doxorubicin + isocyclophosphamide + radiotherapy	12	I
Connor, 2020	55	A	Doxorubicin + isocyclophosphamide	3月R, 12月I	I
Zheng, 2022	23	A	N	12	I
	29	A	N	4	I
Nakra, 2023	52	A + B	N	0.5	I
Kord, 2020	46	N	Gemcitabine + docetaxel	3	N
Wei, 2024	52	A + B	Doxorubicin + isocyclophosphamide	9	S
Wei, 2024	34	A	N	N	N
Baa, 2021	62	A + B + C + D + F	Gemcitabine + paclitaxel	3	S
Yan, 2022	30	A + B + C	Doxorubicin + gemcitabine	4	S
BiYan, 2024	30	G	N	6	S

A = total hysterectomy + bilateral adnexectomy, B = pelviclymph node dissection, C = salpingo-oophorectomy, D = appendectomy, E = pelvic and low para-aortic lymph node dissection, F = retroperitoneal lymph node dissection, G = total uterus + bilateral fallopian tubes, I = die, N = unprecedented, R = recur, S = survive.

For chemotherapy, most of the patients with uterine sarcoma were commonly treated with a combination of docetaxel and gemcitabine or doxorubicin monotherapy. However, the number of SDUS cases was extremely low. Among these 18 patients, 11 patients did not receive supplemental chemotherapy. Two patients were treated with doxorubicin and cyclophosphamide. Two patients were treated with epirubicin and cyclophosphamide, 1 patient was treated with gemcitabine and paclitaxel regimen, 1 patient was treated with doxorubicin and gemcitabine regimen, and there was no significant difference in prolonging the patient’s survival with these chemotherapeutic regimens. Studies are rare, and appropriate treatment regimens cannot be obtained from these cases alone. Hence, the chemotherapy regimens for patients with ovarian tumors with SMARCA4 expression deletion have a specific reference value. According to relevant literature, there are approximately 400 reported cases of SCCOHT.^[8]^ Its chemotherapeutic regimen is diverse and primarily based on platinum, among which VPCBAE (vincristine, cisplatin, cyclophosphamide, bleomycin, doxorubicin, and etoposide) is the most commonly used treatment regimen. In addition, the addition of paclitaxel analogues to this chemotherapeutic regimen may also be of interest.^[9]^ In the case described here, the patient was treated with a platinum-based chemotherapy regimen in combination with docetaxel and epirubicin.

As for radiotherapy, Kurokawaet al^[10]^ reported a case of postoperative SDUS patients who developed liver metastases. Low-dose radiation therapy was used to improve symptoms and prolong survival, suggesting that SDUS may have specific sensitivity to radiotherapy and also that palliative radiotherapy seems to be an effective treatment modality for SDUS patients who develop metastases as well. There is growing research on immunotherapy and targeted therapies for sarcomas. Inhibitors of PD L1, EZH2, and CDK4/6 inhibitors have potential therapeutic value.^[11]^ EZH2 inhibitors have good efficacy for SCCOHT.^[12]^ This provides a promising theoretical basis for exploring the potential of immunotherapy for SDUS as well.

## 4. Conclusion

In conclusion, SDUS is a rare and highly malignant tumor, typically presenting with symptoms such as abnormal uterine bleeding or abdominal pain. There are no specific diagnostic biomarkers, and definitive diagnosis relies on pathological examination. Radical surgical resection combined with postoperative adjuvant radiotherapy can help prolong patient survival. However, whether supplemental postoperative radiotherapy can meaningfully improve overall survival rates requires further study. The limitation of this case was that the follow-up time is relatively short, only up to 3 rounds of chemotherapy after surgery, and the patient’s subsequent condition still needs to be followed up. In clinic, especially for younger women with uterine sarcoma and abnormal uterine bleeding, gynecologic oncologists should consider the possibility of SDUS. The rarity of SDUS cases makes it challenging to establish evidence-based treatment protocols.

## Acknowledgments

We thank the First Affiliated Hospital pathologists with Nanjing Medical University for offering figures and diagnoses of the disease.

## Author contributions

**Conceptualization:** Ying Sun, Huihua Dai.

**Data curation:** Ying Sun.

**Formal analysis:** Ying Sun.

**Supervision:** Ying Sun, Huihua Dai.

**Writing – original draft:** Yu Wang.

**Writing – review & editing:** Ying Sun, Yuxin Jiang, Xing Chen, Huihua Dai.
